# Automated machine learning model for fundus image classification by health-care professionals with no coding experience

**DOI:** 10.1038/s41598-024-60807-y

**Published:** 2024-05-06

**Authors:** Lucas Zago Ribeiro, Luis Filipe Nakayama, Fernando Korn Malerbi, Caio Vinicius Saito Regatieri

**Affiliations:** 1 Department of Ophthalmology and Visual Sciences, Federal University of São Paulo, São Paulo, SP Brazil; 2 Massachusetts Institute of Technology, Institute for Medical Engineering and Science, Cambridge, MA USA

**Keywords:** Eye diseases, Retinal diseases

## Abstract

To assess the feasibility of code-free deep learning (CFDL) platforms in the prediction of binary outcomes from fundus images in ophthalmology, evaluating two distinct online-based platforms (Google Vertex and Amazon Rekognition), and two distinct datasets. Two publicly available datasets, Messidor-2 and BRSET, were utilized for model development. The Messidor-2 consists of fundus photographs from diabetic patients and the BRSET is a multi-label dataset. The CFDL platforms were used to create deep learning models, with no preprocessing of the images, by a single ophthalmologist without coding expertise. The performance metrics employed to evaluate the models were F1 score, area under curve (AUC), precision and recall. The performance metrics for referable diabetic retinopathy and macular edema were above 0.9 for both tasks and CFDL. The Google Vertex models demonstrated superior performance compared to the Amazon models, with the BRSET dataset achieving the highest accuracy (AUC of 0.994). Multi-classification tasks using only BRSET achieved similar overall performance between platforms, achieving AUC of 0.994 for laterality, 0.942 for age grouping, 0.779 for genetic sex identification, 0.857 for optic, and 0.837 for normality with Google Vertex. The study demonstrates the feasibility of using automated machine learning platforms for predicting binary outcomes from fundus images in ophthalmology. It highlights the high accuracy achieved by the models in some tasks and the potential of CFDL as an entry-friendly platform for ophthalmologists to familiarize themselves with machine learning concepts.

## Introduction

The application of artificial intelligence (AI) technologies in ophthalmology has grown exponentially in the last decade^1,2^. This expansion can be attributed to the combination of advancements in machine learning performance in the last years and the appropriate characteristics found in ophthalmology for such applications^3^. Ophthalmology provides a suitable field for the diagnosis and evaluation of ocular diseases using non-invasive imaging exams, particularly fundus photographs and optical coherence tomography. The main application of AI in eye diseases includes screening for diabetic retinopathy (DR)^4^, age-related macular degeneration^5^, glaucoma^6^, and keratoconus^7^.

In addition to facilitating direct examination for the early detection of sight-threatening diseases, the assessment of the retina also allows for the correlation with systemic conditions, including cardiovascular risk^8^, Alzheimer’s disease^9^, and even sex identification through fundus photographs^10^.

The machine learning models have reached similar or even better performance than experienced retinal specialists for fundus photography classification tasks such as DR screening^4^. However, coordinating the requirements to develop machine learning algorithms in healthcare is challenging due to limited resources and a restriction on access to talented teams of data experts for some research groups, especially in low-and-middle income countries, such as Brazil. Besides, there is an important disparity bias in algorithm development and dataset distribution between populations in developed and developing countries, including sociodemographic disparities^11–13^.

To address these challenges, automated machine learning (AutoML) offers a promising solution. AutoML is a collection of tools and techniques for optimizing model development by automating the selection of optimal network architectures, pre-processing methods, and hyperparameter optimization. As these platforms continue to mature, the automation of these processes may reduce the need for programming experience required to design such models. Some platforms offer a code-free deep learning approach, which is even more accessible to clinicians or researchers without coding expertise.

Despite the potential, there are a limited number of studies evaluating AutoML platforms in ophthalmology, which have found high accuracy^7,10,14–17^. Google AutoML and Amazon Rekognition were the leading platforms in previous multi-platform study^18^. Besides, Google recently integrated the previous Google AutoML Vision platform into the Google Vertex AI, which has been promised as the new generation of Google’s AutoML for images.

We aimed to assess the feasibility of AutoML models to predict binary outcomes from fundus images comparing two distinct online-base code-free deep learning (CFDL) platforms.

## Results

Table 1 and 2 provide a summary of the image characteristics and patient demographics. The BRSET dataset is predominantly composed of females, accounting for 61.81%, with a mean age of 23.43 ± 8.56 years.

**Table 1:** The performance of the models evaluating referable diabetic retinopathy classification and macular edema.

Dataset	Labels		Performance			Platform
			F1 score	Precision	Recall	
Messidor-2	No DME	DME				
	1593	151	0.919	0.924	0.915	Amazon
	1593	151	0.977	0.977	0.977	Google
	NRDR	RDR				
	1279	465	0.907	0.937	0.885	Amazon
	1279	465	0.937	0.937	0.937	Google
BRSET	NRDR	RDR				
	1883	810	0.935	0.943	0.928	Amazon
	1883	810	0.963	0.963	0.963	Google
	No DME	DME				
	2334	359	0.914	0.922	0.907	Amazon
	2334	359	0.967	0.967	0.967	Google

*DME* diabetic macular edema, *NRDR* non-referable diabetic retinopathy, *RDR* referable diabetic retinopathy.

**Table 2:** The performance of the models evaluating multi-label tasks using the BRSET dataset images.

Task	Labels		Performance			Platform
			F1 Score	Precision	Recall	
Laterality	Right eye	Left eye				
	7056	7221	0.992	0.992	0.992	Amazon
	7056	7221	0.992	0.992	0.992	Google
Age	< 65 yo	≥ 65 yo				
	5960	3898	0.858	0.857	0.858	Amazon
	5960	3898	0.88	0.88	0.88	Google
Genetic sex	Male	Female				
	5451	8826	0.779	0.723	0.845	Amazon
	5451	8826	0.777	0.777	0.777	Google
Normality	Normal	Abnormal				
	7291	6986	0.837	0.812	0.868	Amazon
	7291	6986	0.833	0.833	0.833	Google
Optic disc cupping	Normal	Enlarged				
	11,361	2916	0.857	0.852	0.862	Amazon
	11,361	2916	0.895	0.895	0.895	Google

**Figure 1:**
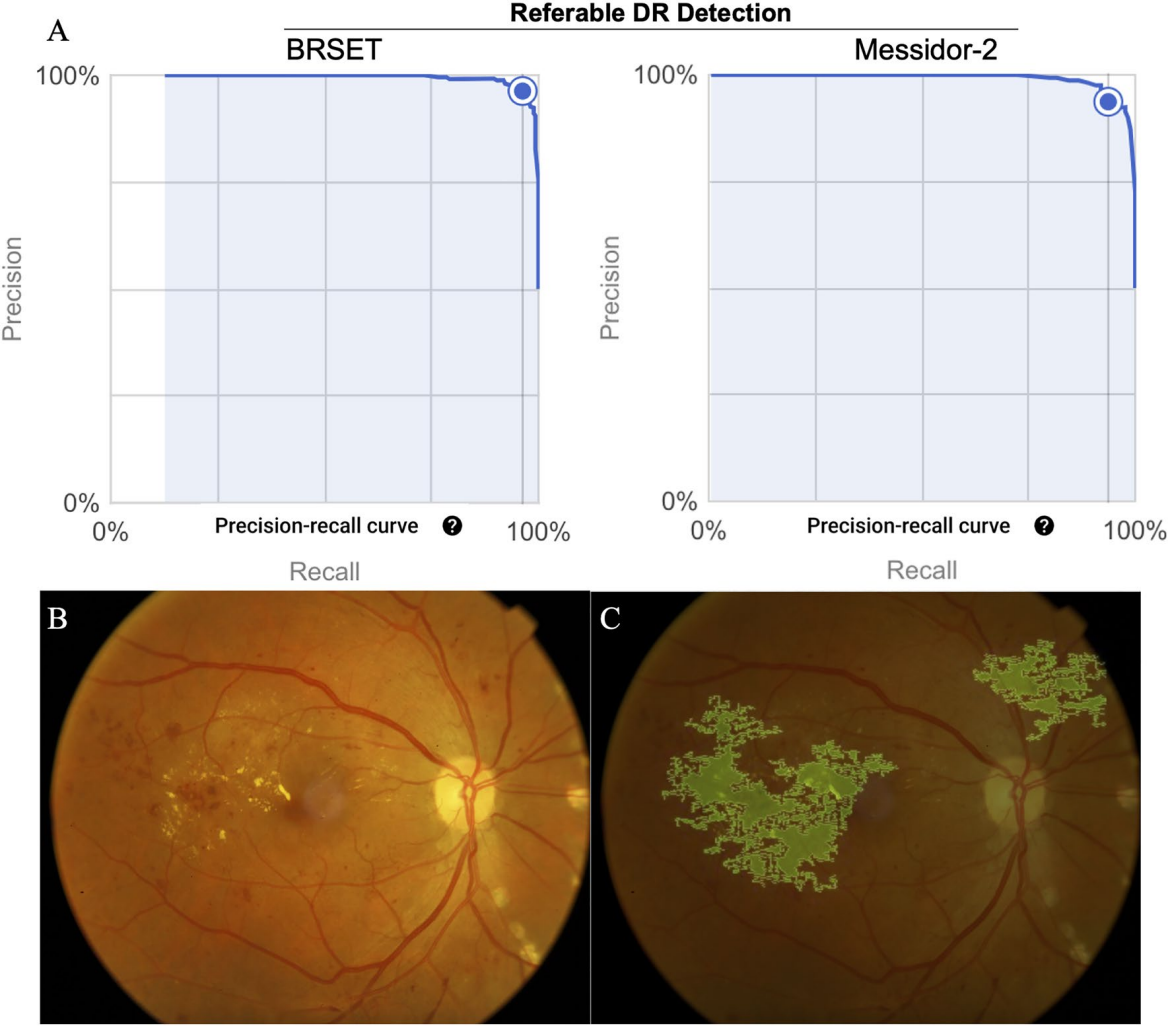
Google AutoML performance report demonstrates precision-recall curves of BRSET and Messidor-2 in detecting referable diabetic retinopathy (**A**) along with the related saliency map, showing the input image (**B**) and the resulting saliency map (**C**).

**Figure 2:**
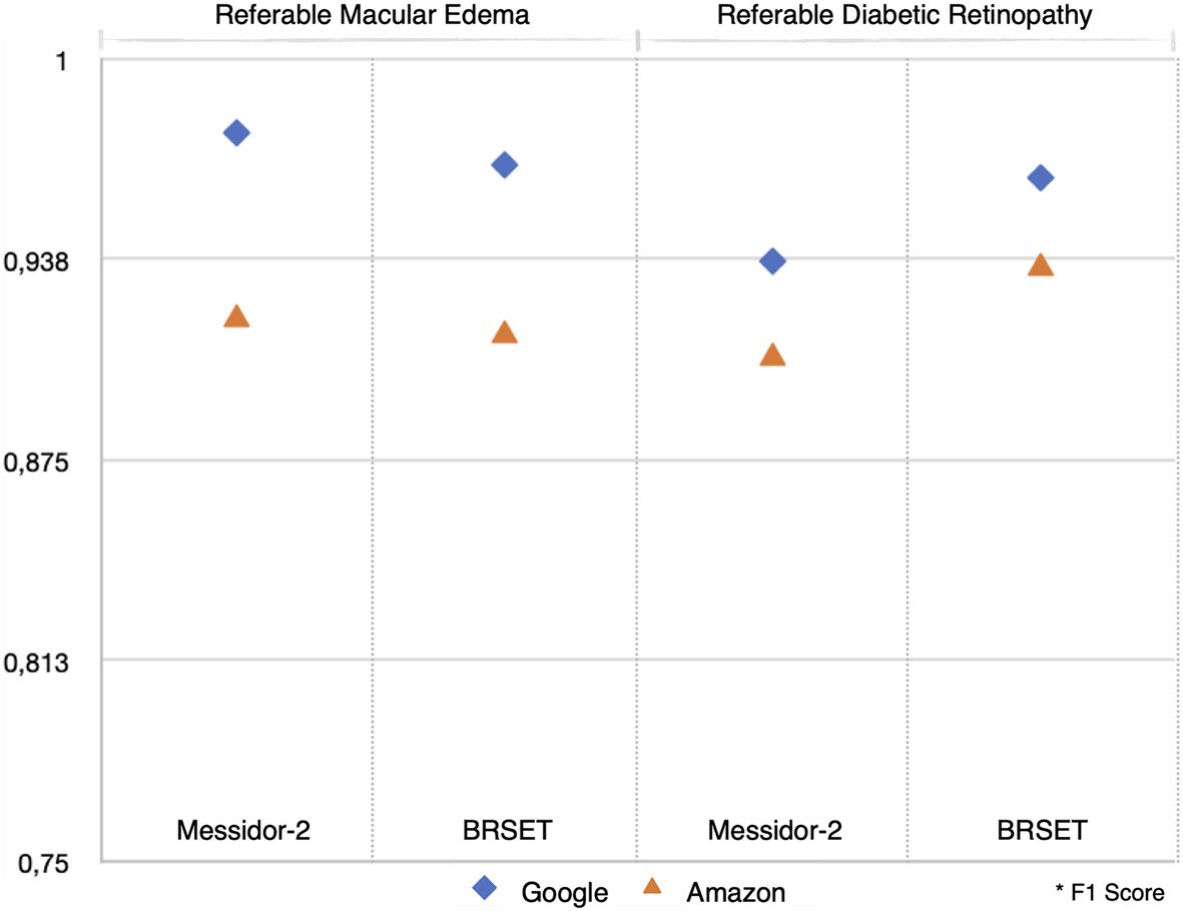
Models performance for diabetic retinopathy, comparing the datasets and platforms.

We assessed the performance of the classification models for non-referable DR (NRDR) vs referable DR (RDR) using Google and Amazon Web Services (AWS) AutoML platforms for binary classification and using Messidor-2 (1744 images) and BRSET (2693 images) datasets. The AWS models obtained an F1 score of 0.907 for Messidor-2 and 0.95 for the BRSET dataset, while the Google Vertex models obtained 0.937 (AUC of 0.992) and 0.963 (AUC of 0.994), respectively (Table 1 and Figs. 1, 2).

The F1 score obtained for the classification of the presence or absence of macular edema using the AWS models were 0.919 for the Messidor-2 and 0.935 for the BRSET, and the Google models were 0.977 (AUC of 0.973) for the Messidor-2 and 0.967 (AUC of 0.986) for the BRSET. (Fig. 2).

The Google platform offers a feature-based explanation for each image in the model, indicating the contribution of each feature to the predictions for a given instance. This explanation generates a heat map overlaying the fundus photo, illustrating the regions that influenced the model’s predictions. Figure 1 showcases an explainable image of RDR, created using the integrated gradients method.

External validation is available only on Google’s platform, which supports batch prediction, and was performed using the IDRiD diabetic retinopathy dataset^19^. The referable models for Google Messidor and Google BRSET demonstrated F1 score; (precision, recall, AUC) of 0.897 (0.829, 0.977, 0.873) and 0.846 (1.0, 0.733, 0.966), respectively. The macular edema models for the Messidor and BRSET demonstrated 0.857 (0.907, 0.812, 0.920) and 0.886 (0.975, 0.812, 0.922), respectively.

After excluding missing labels for the multi-classification tasks using the BRSET. We included 14,277 images for laterality, sex identification, normality and optic disc cupping, and included 7886 images for age groups identification.

**Figure 3:**
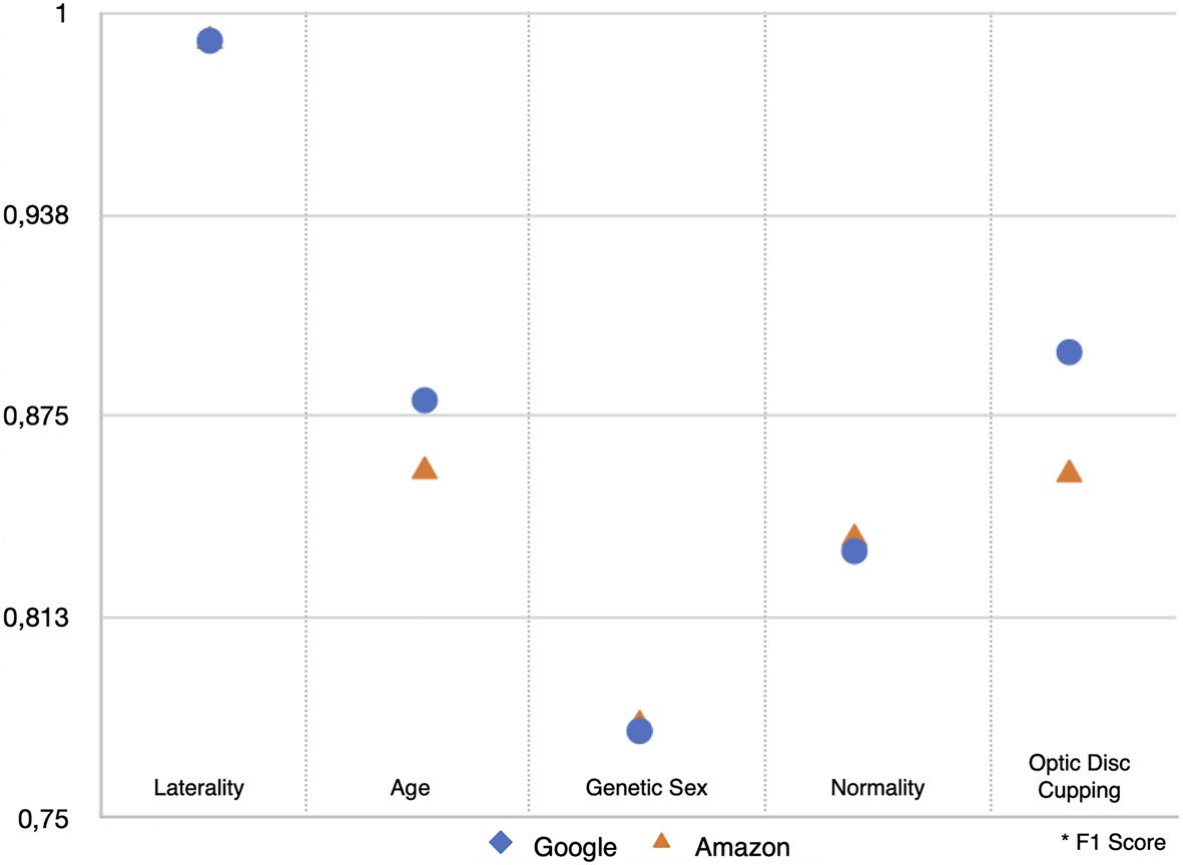
Models performance for multi-task classification using BRSET dataset, and comparing the platforms.

The performance of multi-classification binary tasks using the BRSET images by the Google Vertex, represented as F1 score (AUC), for laterality was 0.992 (0.994), age was 0.88 (0.942), sex was 0.777 (0.849), optic disc cupping was 0.895 (0.958) and normality was 0.833 (0.915). The performance for the AWS models, represented as F1 score, for laterality was 0.992, age was 0.858, sex was 0.779, optic disc cupping was 0.857 and normality was 0.837 (Table 2 and Fig. 3).

Table 1 and 2 summarizes the performance and distribution sets for all the tasks.

## Discussion

The performance metrics for detecting referable DR tasks were above 0.9 on both datasets and CFDL platforms. The highest accuracy was achieved with the Google Vertex models over the AWS models, and the highest accuracy was observed with the BRSET. Previous study from Korot in 2021^18^, which was the first to demonstrate the use of multiple datasets and platforms in ophthalmology, found models with lower performance using the same Messidor-2 dataset tasks on Google and AWS CFDL platforms, with F1 scores of 0.848 and 0.885, respectively. This could highlight that the platforms are continually updating to achieve more optimized predictions. Google’s website suggests improved performance with Vertex AI, accompanied by migration tutorials from AutoML Vision to Vertex AI.

Although the Messidor-2 dataset has been used by several groups, there are different allocations of referable DR images^20,21^ and usually used for external validation, not allowing direct comparison with previous traditional machine learning studies^22,23^.

The metrics related to the detection of referable DME using both datasets were also above 0.9, with the highest accuracy achieved with the BRSET and Google Vertex (AUC of 0.986). Previous study obtained an AUC of 0.958 using non automated machine learning^24^.

The results of the multi-classification tasks showed heterogeneity depending on the evaluation point, as expected^25^. Both platforms achieved similar F1 scores for most tasks, with an performance above 0.8 for laterality, age, optic disc cupping, and normality. Google performed significantly better than AWS for optic disc cupping (0.895 × 0.857) and age grouping (0.88 × 0.858). We opted to perform a simple task such as differentiating between right and left eye to check the platforms’ functionality within the BRSET. Both platforms achieved an equal F1 score of 0.992 for this task.

Previous studies have demonstrated high performance of machine learning models for genetic sex identification, achieving an AUC of 0.93 using Google AutoML trained with the 173,819-image UK Biobank dataset^10^. One possible explanation for the lower accuracy observed in our study is the significantly smaller dataset used (BRSET, with 14,277 images), which resulted in the highest AUC of 0.849 with the Google platform.

The presence of imbalanced distribution between classes and overfitting is a concern of any machine learning algorithm^26^. This issue affects the accuracy of models and especially real-world performance^27^. In our study, the DR tasks were the most imbalanced ones, and the external validation of the Google Vertex models revealed good performance with the IDRiD dataset for both referable and macular edema tasks. Additionally, in line with what most previous studies have done, we chose not to divide eyes from the same patient into different subsets, although it could introduce bias into our findings.

Despite being known as code-free platforms, training AutoML models still requires the management of large image datasets and working with “.csv” files. It is an interesting way for ophthalmologists to become more familiar with machine learning concepts and challenges. Both the platforms tested offer feasible research grant programs that allow using and testing the platform, for even initial researches, with free credits. The future of healthcare is expected to be significantly influenced by advancements in AI, and CFDL serves as an entry-friendly platform for learning fundamental concepts.

Our study demonstrates that ophthalmologists with no coding experience can construct high-accuracy CFDL models using a locally developed dataset^28^ and compare the performance of two different platforms. Although this facilitates access to machine learning for small research groups and non-experts, it remains crucial to utilize appropriate datasets, as smaller or lower-quality datasets may introduce greater bias^29^.

The clinical application of AutoML models still faces several barriers, including high costs, the difficulty in extracting useful information from the “black box” nature of the models^30^, and the fact that platforms are not yet suitable for approval by regulatory agencies at the level required for clinical trials^16^. Furthermore, there are currently no real-world applications of AutoML models in ophthalmology.

Large language models (LLMs), popularized by the emergence of ChatGPT and already tested in ophthalmology using question banks^31,32^, could play an important role in assisting the development of machine learning models for non-experts, such as coding^33^ or at least in helping to interpret the steps required. Even when using CFDL platforms, which are designed to be user-friendly, there is still a requirement to manipulate large sets of images and work with “.csv” and “.jsonl” files. In our opinion, LLMs can provide assistance at each step of using most AutoML platforms. It is important to use it with caution, and verify their outputs with external methodologies^34^.

Limitations of some CFDL platforms, such AWS used in this study, is the absence of batch prediction, which makes external validation of the models challenging.

In conclusion, we demonstrate the feasibility of using CFDL by retina specialists without coding experience, reaching similar performance as previous studies, particularly when evaluating diabetic retinopathy tasks. We emphasize the high accuracy achieved by the models in specific tasks and the potential of CFDL as a user-entry-friendly platform for ophthalmologists to become acquainted with machine learning concepts.

## Methods

The study was approved by the Federal University of São Paulo (UNIFESP) ethics committee (CAAE 49171021.6.0000.5505).

### Data source and data preparation

We included two distinct publicly available datasets for deep learning model development to evaluate referable diabetic retinopathy (DR): Messidor-2 and BRSET. The Messidor-2 is a well-known ophthalmology dataset that consists of 1744 fundus photographs in .png format taken with a Topcon TRC NW6 fundus camera (Tokyo, Japan), labeled according to the DR ICDR protocol (grades 0–4) and diabetic macular edema classification^35^. The Brazilian multilabel ophthalmological dataset (BRSET) is a multi-labeled ophthalmological dataset, developed by our research group, which consists of 16,266 fundus photographs in .jpg format from 8524 Brazilian patients taken with a Nikon NF505 (Tokyo, Japan) and a Canon CR-2 (Melville, USA)^28^.

Both dataset labels were regrouped considering the ICDR protocol DR classification into NRDR, which includes absence or mild retinopathy, and RDR, which includes moderate or worse retinopathy. Only images from diabetic patients were included from BRSET, to become comparable to the Messidor-2 dataset, resulting in a total of 2693 images.

### Code-free deep learning platforms

Two online-based CFDL platforms were used to develop deep learning models from the datasets above, which were Google Cloud AutoML Vertex AI and AWS Rekognition Custom Labels. The images were not pre-processed before upload to the platforms and the models were created by a single ophthalmologist with no coding experience.

### Outcomes

The multi-label tasks included only the BRSET dataset and we opted to exclude from analysis the images with low quality parameters (focus, illumination, image field and artifacts) provided by the dataset labels^28^. So, 14,277 images were included and the multi-label two-class parameters grading: normality, laterality, sex identification, age grouping, and optic disc cupping referral.

Images were randomized into the following: training (80%), optimization/validation (10%), and testing (10%), with the distribution sets manually configured. The images were pre-processed and the platform used pre-trained models as a starting point, optimizing parameters and fine-tuning to achieve the best algorithm for the sample data.

### Models outcome and performance evaluation

The CFDL platform reveals the performance obtained by the created algorithm for the used dataset. The Google AutoML evaluates performance as average precision (calculated by the area under the precision-recall curve), precision (positive predictive value) and recall (sensitivity), using a confidence threshold of 0.5. The AWS Rekognition measures performance as F1 score, average precision and overall recall. The F1 score combines precision and recall into one metric by calculating the harmonic mean between those two, and usually performs better on imbalanced datasets^36^.

The F1 score was calculated for the Google platform using precision and recall, to be comparable with the AWS metrics.

External validation was performed for the diabetic retinopathy models using the IDRiD dataset^19^ on the batch prediction section of the Google platform. Outcome metrics were F1 score, AUC, precision and recall.

## Data Availability

The primary datasets included in this study are publicly available (Messidor-2^22,35^, BRSET^28^ and IDRiD^19^).
